# Spatial clustering and risk factors of malaria infections in Ratanakiri Province, Cambodia

**DOI:** 10.1186/1475-2875-13-387

**Published:** 2014-09-30

**Authors:** Vincent Sluydts, Somony Heng, Marc Coosemans, Karel Van Roey, Charlotte Gryseels, Lydie Canier, Saorin Kim, Nimol Khim, Sovannaroth Siv, Vanna Mean, Sambunny Uk, Koen Peeters Grietens, Sochantha Tho, Didier Menard, Lies Durnez

**Affiliations:** Institute of Tropical Medicine, Antwerp, Belgium; National Center for Parasitology, Entomology and Malaria Control, Phnom Penh, Cambodia; Institut Pasteur du Cambodge, Phnom Penh, Cambodia; School of International Health Development, Nagasaki University, Nagasaki, Japan; Partners for Applied Social Sciences (PASS) International, Tessenderlo, Belgium; Department of Biomedical Sciences, University of Antwerp, Antwerp, Belgium

## Abstract

**Background:**

Malaria incidence worldwide has steadily declined over the past decades. Consequently, increasingly more countries will proceed from control to elimination. The malaria distribution in low incidence settings appears patchy, and local transmission hotspots are a continuous source of infection. In this study, species-specific clusters and associated risk factors were identified based on malaria prevalence data collected in the north-east of Cambodia. In addition, *Plasmodium falciparum* genetic diversity, population structure and gene flows were studied.

**Method:**

In 2012, blood samples from 5793 randomly selected individuals living in 117 villages were collected from Ratanakiri province, Cambodia. Malariometric data of each participant were simultaneously accumulated using a standard questionnaire. A two-step PCR allowed for species-specific detection of malaria parasites, and SNP-genotyping of *P. falciparum* was performed. SaTScan was used to determine species-specific areas of elevated risk to infection, and univariate and multivariate risk analyses were carried out.

**Result:**

PCR diagnosis found 368 positive individuals (6.4%) for malaria parasites, of which 22% contained mixed species infections. The occurrence of these co-infections was more frequent than expected. Specific areas with elevated risk of infection were detected for all *Plasmodium* species. The clusters for Falciparum, Vivax and Ovale malaria appeared in the north of the province along the main river, while the cluster for Malariae malaria was situated elsewhere. The relative risk to be a malaria parasite carrier within clusters along the river was twice that outside the area. The main risk factor associated with three out of four malaria species was overnight stay in the plot hut, a human behaviour associated with indigenous farming. Haplotypes did not show clear geographical population structure, but pairwise Fst value comparison indicated higher parasite flow along the river.

**Discussion:**

Spatial aggregation of malaria parasite carriers, and the identification of malaria species-specific risk factors provide key insights in malaria epidemiology in low transmission settings, which can guide targeted supplementary interventions. Consequently, future malaria programmes in the province should implement additional specific policies targeting households staying overnight at their farms outside the village, in addition to migrants and forest workers.

**Electronic supplementary material:**

The online version of this article (doi:10.1186/1475-2875-13-387) contains supplementary material, which is available to authorized users.

## Background

Globally, malaria incidence has decreased by 29% between 2000 and 2012, while malaria specific mortality rates have been reduced by 45% over the same period [1]. Increasingly more and more countries are therefore expected to move from a malaria control phase to an elimination phase. This shift requires a re-orientation of the intervention strategies to combat the disease [2, 3]. In a control programme, the main objective is to reduce the disease burden, while the complete and continuous ending of local transmission is required in the elimination phase [2, 4]. At all levels of endemicity, but particularly in areas of low transmission, malaria infections tend to cluster in hotspots which remain a continuous source of infection [5, 6]. Hence, making it increasingly important to target these foci of malaria transmission [1, 2, 4, 6].

Since 2000, Cambodia has reported a 75% decrease in microscopically-confirmed malaria cases in the public sector [7]. This indicates that Cambodia, which reported an average incidence of 3.1/1,000 cases in 2012 [8], is progressing towards a pre-elimination phase as intended in their national strategic plan for the elimination of malaria [8]. However, the complexity of the malaria epidemiology in Cambodia and surrounding countries might hamper further progress towards elimination. The Thai-Cambodian border for example, is the epicenter of the emergence of multidrug resistant parasites including resistance to artemisinin derivatives [9, 10]. Furthermore a diverse community of *Anopheles* species (or species complexes) showing heterogeneous biting behaviour occur in the area, of which at least four are capable of transmitting the infection (i.e. sporozoites in the salivary glands) [11, 12]. Also, G6PD deficiency is known to be prevalent among the Cambodian population possibly jeopardizing the safe routine use of primaquine to reduce *P. falciparum* transmission or to prevent *Plasmodium vivax* relapses [13]. This illustrates that several hurdles remain to be taken to improve the existing malaria control programme. At present the National Malaria Programme tends to focus on the containment of *P. falciparum* artemisinin resistance and on scaling up interventions country-wide. In order to reach the level of endemicity that would enable the country to move into a pre-elimination phase, the National Malaria Programme needs to implement supplementary intervention strategies for tackling the heterogeneous nature of the disease in Cambodia. Targeting hotspots could be a highly efficient way to reduce malaria transmission intensities locally [6].

The study presented here aims to characterize malaria clusters in the outmost north-eastern province of Cambodia using a combination of advanced molecular diagnostic tools [14] and an efficient, rapid, and large scale random sampling of the population. This allows investigating whether the occurrence of different *Plasmodium* species has a tendency for clustering throughout space. In addition, several potential risk factors and individuals’ characteristics have been associated with the prevalence of parasite carriers. Furthermore the *P. falciparum* genetic diversity was studied and linked with the geographical patterns found.

The entire study was considered a pilot for a large-scale intervention whereby the effect of topical repellents, in addition to the use of long-lasting insecticidal nets was assessed. Topical repellents, as part of the forthcoming intervention, were not yet distributed during the period of this study. The entire intervention, conducted between March 2012 and December 2013, was registered as NCT01663831.

## Methods

### Field setup and study design

The study was conducted in Ratanakiri province located in the north-east of Cambodia. Diagnostic-based incidence rate in Ratanakiri was estimated to be 22.2 per 1,000 inhabitants in 2007, which is among the highest in the country [15]. Malaria transmission tends to peak during the rainy season (June – October), while it is generally acknowledged that migrants and forest workers are among the population most at risk to *Plasmodium* infections in the greater Mekong region [15].

A total of 117 villages out of 240 were selected based on malaria incidence records obtained from the Cambodian National Malaria Control Programme and collected between 2009 and 2011 [16]. Villages with the highest incidence were selected. The average number of inhabitants per village was 468 (range: [53-3228]). A random sample of 65 individuals per village, excluding children below two years of age, was extracted based upon the population census of 2011. Samples were collected during the dry season. Between 31 January 2012 and 24 February 2012, five teams of four people visited each village for two consecutive days. The aim was to collect at least 50 samples in each village over a two-day period. When a team indicated to be unable to reach sufficient people during the first day, an extra list of 15 randomly selected individuals was provided on the second sampling day. Blood samples were collected by finger prick and immediately stored in labeled 96-well plates and on filter paper as previously described in [14]. Each individual received a unique code number and malariometric data were recorded using a standardized questionnaire (Additional file 1). Gender, age and ethnicity were recorded as specific individual variables. Axillary temperature was measured and the participants were asked for their history of fever over the past 48 hours. Individuals with fever or other malaria related symptoms were checked by a rapid diagnostic test (CareStart™ Malaria, pLDH/HRP2 COMBO (PAN/Pf)). Malaria positive cases were treated by dihydroartemisinin plus piperaquine combination (Duo-Cotecxin®: Beijing Holley – Cotec Pharmaceutical CO., Ltd, Being, China), according to the national treatment guidelines. Behaviour related to bed net use, sleeping and waking times, overnight stays in homes at the farms located outside the village (plot huts) and overnight stays during activities in the forest were recorded in a subsequent form (Additional file 1 for detailed questions).

### Malaria PCR detection

Blood samples were transported in cooled boxes by a taxi service to Institut Pasteur in Phnom Penh within 24 h. As previously described [14], DNA extraction followed by a two-step Real-time PCR assay was performed to detect malaria parasites. Briefly, in a first step, each blood sample was screened using genus-specific primers for malaria infection. In a second step, only positive samples were tested for *Plasmodium* species identification using species-specific primers for the four main human malaria species (*Plasmodium falciparum*, *Plasmodium vivax*, *Plasmodium ovale*, *Plasmodium malariae*). Results were available 48 hours after the blood collection took place, allowing for rapid assessment and treatment of malaria positive cases.

### Statistical analysis

#### Data entry

All survey data were double entered and cross-checked into an Access database with preprogrammed forms to minimize data-entry errors. Each individual was blinded by a 10 digit code and the PCR results were digitally merged with the survey data based on this unique code.

#### Association between pure and mixed malaria infections

Frequency data on the number of pure and mixed infections were analysed using a log-linear model as described in [17]. Statistical non-independence between different malaria species was tested with a likelihood ratio test comparing a reduced model including single parasite species with one including all two-way interactions. Two-way associations were tested by comparison of a reduced model including all single parasite species and the same model including the specific two-way interaction. The reduced model in the three-way case had all single and double/multiple species infections and was compared to a model with a specific three-way interaction. All test statistics were based on the likelihood ratio tests and associated p-values were reported [17].

#### Risk factor analysis

The association of individual’s characteristics, potential risk behaviour and the malaria prevalence of *Plasmodium* species was investigated by fitting generalized linear mixed models with a binomial error distribution to the data. Potential clustering of malaria prevalence at the village level was taken into account by incorporating village as a random effect in the model. All 95% confidence intervals (CI) and odd ratios (OR) were calculated as the exponentials of the fixed effects parameters.

Firstly, all explanatory variables were tested in a univariate model. Age, gender, axillary temperature, ethnicity (Khmer versus ethnic minority) and sleeping/waking-up times were considered as individual characteristics. Overnight stays at the farms and in the forest and use of a bed net were considered potential risk factors. Statistical significance was evaluated based upon a likelihood ratio test and associated p-values were reported.

In a subsequent step a multivariable random effects logistic regression was fitted and the Akaike Information Criterion (AIC) was used to select the model which best fitted the data [18]. As a starting point the variables showing a potential association (p < 0.10) with malaria risk in the univariate model were included with all plausible two- and three -way interactions. Given that this study did not aim to search for a clinical proxy for any of the malaria species, axillary temperature was omitted from the multivariate analysis. Model selection was performed using a Laplace approximation, while the parameter estimates of the final model were obtained using an Adaptive Gaussian Quadrature with 20 quadrature points.

Independent categorical variables were screened for multicollinearity with a Cramér’s V statistic. In case two variables showed signs of correlation (V > 0.7), the variable least associated with the response variable was omitted.

#### Spatial statistics

The spatial software SaTScan (v9.1.1 64-bit), which was demonstrated to be the preferred software tool under low relative risk settings [19], was used to detect spatial clusters of infected individuals. In SaTScan clusters were detected by gradually scanning a circular window across space, noting the number of observed and expected observations inside the window at each location. Multiple different window sizes were used up to circles containing a maximum of 50% of the population at risk (details can be found in the SaTScan manual [20]. Clusters were assessed based on 999 Monte Carlo simulations to determine the probability of observed prevalence of PCR positive individuals for each *Plasmodium* species being due to chance relative to expected prevalence under the null hypothesis of no clustering. The window with the maximum likelihood was the most likely cluster, and a p-value was assigned to this cluster. Note that the result of this statistical method provides a cluster including data points with an elevated risk to infection, but also includes areas where no observations were done. Hence the circles drawn to represent those clusters are only indicative to the true area under which an elevated risk can be expected. The relative risk reported for each observed cluster was the estimated risk within the cluster divided by the estimated risk outside the cluster [20].

#### *Plasmodium falciparum*genotyping

Genotyping was performed as described in [21], with modifications. Seven SNPs were assessed using a PCR-LDR-FMA (PCR-Ligase Detection Reaction-Fluorescence Microspheres Assay). Protocols, PCR/nested PCR primer sequences, and LDR probe sequences (assays No. 8, 9, 12, 16, 19, 20 and 24) are available in [22]. Nearby villages were pooled together in 20 clusters based on a visual inspection of the distribution of villages in the province. Average distance between the cluster centroids was 11.3km and ranged between 6.8 and 19.2 km. For each *P. falciparum* isolates, SNPs were concatenated in one sequence (=barcode), representing the genome diversity, grouped in clusters. Genetic diversity for each cluster was assessed by Nei’s unbiased expected heterozygosity (He) from haploid data and calculated as He = [n/(n-1)][1-p_i_] (n = the number of isolates sampled; p_i_ = the frequency of the i^th^ allele [23]. Population genetic differentiation by clusters was measured using Wright’s F statistics [24]. Population genetic parameters were computed with FSTAT software, v2.9.4 [25].

#### Ethical clearance

The study protocol was reviewed and approved by the Cambodian National Ethics Committee on Health Research (Approval 265 NECHR), the Institutional Review Board of the Institute of Tropical Medicine Antwerp (Approval IRB/AB/ac/154) and the Ethics Committee of the University of Antwerp (Approval B300201112714). Gatekeepers provided informed written consent for the participation of their village. The survey participant or his/her parents or guardian provided informed written consent for individual participation.

## Results

### Summary statistics and association between parasites

During the 24-day survey period, all 117 villages were visited. Blood samples from 5793 individuals were collected using a standard finger prick method. An extra list of randomly chosen individuals was provided in 43 out of 117 villages, resulting in a response rate of 70% (5793/8250). Malaria prevalence by PCR was 6.35% (368 cases out of 5793 tested individuals). *Plasmodium falciparum* (139/368) and *P. vivax* (139/368) were the most common species, while 22.3% (82/368) were mixed infections, mainly *P. falciparum*/*P. vivax* co-infection (56/368) (Table 1). No single *P. ovale* infection was detected. A quadruple co-infection was observed in one young [age =13 y] female. Her malariometric data indicated that she did not use a bed net, and that she had stayed overnight in a plot hut on a farm outside the village during the last month. Associations between all double co-infections observed were found to be positive and significantly higher as expected (Table 1). One three-way association (*P. falciparum*/*P. vivax*/*P. ovale*) was found to be significant.

**Table 1:** **Overview of the prevalence of malaria positive samples per species with indication of the direction of the association in case of a mixed infection, Ratanakiri province, Cambodia 2012**

Species	Frequency	% Prevalence	Association	p-value
Pf	139	2.399	NA	NA
Pv	139	2.399	NA	NA
Pm	8	0.138	NA	NA
Po	0	0.000	NA	NA
PfPv	56	0.967	+	<0.001
PfPm	5	0.086	+	<0.001
PfPo	1	0.017	+	0.012
PvPm	3	0.052	+	<0.001
PvPo	11	0.190	+	<0.001
PmPo	0	0.000	+	0.041
PfPvPm	4	0.069	NS	0.091
PfPvPo	1	0.017	-	0.003
PfPmPo	0	0.000	NS	0.430
PvPmPo	0	0.000	NS	0.909
PfPvPmPo	1	0.017	NA	NA
Total Negative	5425	93.648		
Total Positive	368	6.352		
Total sample	5793			

Pf = *P. falciparum*, Pv = *P. vivax*, Pm = *P. malariae*, Po = *P. ovale*.

NS = Not Significant, NA = Not Applicable.

More females participated in the study as compared to males (52.7% females, 47.3% males, X^2^ = 34.24, df = 1, p < 0.001). Self-reported use of bed nets was 92.4%. Among bed net users, 77.5% stated to sleep under a long-lasting insecticidal net (LLIN) provided by the National Malaria Programme, while the remaining 22.5% slept under a net bought on the market. More than half of the individuals (56.2%) reported to have stayed overnight at their plot hut the month prior to the interview, while this was only 16% for overnights in the deep forest. A gender bias was observed for individuals staying overnight in the forest (27.3% females, 72.7% males; Χ;^2^ = 379.76, df = 1, p < 0.001), but not at the plot hut (50.7% females, 49.3% males, Χ;^2^ = 1.0865, df = 1, p = 0.297). 75% of the participants declared to wake up before 6 a.m. and 75% to go to bed before 9 p.m.

### Risk factor analysis

**Figure 1:**
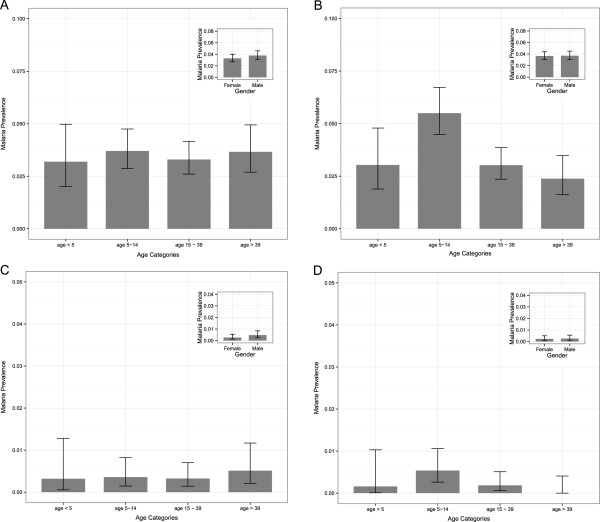
**Prevalence of** ***Plasmodium*** **species detected by age groups with inset of the gender specific prevalence.** Panels: **A**: *Plasmodium falciparum*, **B**: *Plasmodium vivax*, **C**: *Plasmodium malariae*, **D**: *Plasmodium ovale*. Error bars indicate 95% confidence intervals.

Univariate analysis of risk factors for malaria infections per species shows similar association trends between each of the malariometric variables and malaria prevalence (Table 2). *Plasmodium* spp. tended to be more prevalent in the age group 5-14, except for *P. falciparum* and *P. malariae* (Figure 1). No differences in malaria prevalence according to gender were found (OR_all_ and 95% CI with respect to males: 0.90 [0.72-1.12], p = 0.33, Figure 1). Ethnic minorities tended to be more at risk for *Plasmodium* infection as compared to the Khmer majority (OR_all_ and 95% CI with Ethnic Minority as reference: 2.81 [0.97-8.14], p = 0.025) and this was demonstrated to be significant in the pooled *Plasmodium* analysis (p = 0.025) and for *P. falciparum* (p = 0.034). Note that no *P. malariae* cases were observed in the Khmer Ethnic population. An increased risk for a *Plasmodium* infection was observed in individuals with an axillary temperature ≥ 37.5 (OR_all_ and 95% CI with <37.5 as reference: 2.04 [1.21 – 3.45]). No significant associations between *Plasmodium* spp. infections and sleeping times were found. Individuals that reported to stay overnight in their plot hut showed an increased risk to infection (OR_all_ with respect to an overnight stay and 95% CI: 1.50 [1.17-1.93], p = 0.0014) and this was found to be significant in the pooled *Plasmodium* analysis and for *P. falciparum* (p < 0.001) and *P. malariae* (p < 0.001). The variables overnight stay in the forest and use of bed net were not found to be significant for any of the *Plasmodium* species. Odds ratios estimates for use of bed net showed a tendency, though non-significant, for a decreased risk of infection for all *Plasmodium* species when a bed net was used (OR_all_ and 95% CI: 0.77 [0.49-1.22], p = 0.2748, Additional file 2).

**Table 2:** **Univariate analysis of risk factors for all** ***Plasmodium*** **species combined and separate for** ***P. falciparum***, ***P. vivax***, ***P. malariae*** **and** ***P. ovale***, **Ratanakiri province**, **Cambodia 2012**

Malariometric variable		*Plasmodium*( *all*)				*P. falciparum*				*P. vivax*				*P. malariae*				*P. ovale*			
Variable	Level	OR	LCL	UCL	p-value	OR	LCL	UCL	p-value	OR	LCL	UCL	p-value	OR	LCL	UCL	p-value	OR	LCL	UCL	p-value
**Age (years)**					**0.0158**				0.8396				< **0.001**				0.8691				**0.0101**
NA = 167	2-5	reference				reference				reference				reference				reference			
	5-14	1.489	0.998	2.220		1.224	0.715	2.094		1.895	1.119	3.210		1.122	0.174	7.228		2.161	0.184	25.452	
	15-39	1.041	0.696	1.557		1.103	0.650	1.874		1.038	0.602	1.789		1.009	0.162	6.289		0.623	0.046	8.451	
	≥40	0.983	0.632	1.528		1.219	0.691	2.150		0.783	0.421	1.454		1.586	0.246	10.239		0.000	0.000	Inf	
**Gender**					0.3252				0.2859				0.8635				0.1787				0.8101
NA = 34	male	reference				reference				reference				reference				reference			
	female	0.896	0.719	1.117		0.856	0.639	1.147		0.976	0.732	1.300		0.550	0.197	1.535		0.876	0.270	2.845	
**Ethnicity**					**0.0246**				**0.0344**				0.1698				0.1913				0.3692
NA = 49	Khmer	reference				reference				reference				reference*				reference			
	Ethnic Minority	2.806	0.968	8.136		3.586	0.799	16.086		2.118	0.611	7.343		-	-	-		0.317	0.025	4.075	
**Axillary Temp**					**0.0098**				**0.0228**				0.0782				0.6108				0.1312
NA = 74	< 37,5	reference				reference				reference				reference				reference			
	≥ 37,5	2.043	1.210	3.451		2.280	1.176	4.419		1.916	0.964	3.808		1.775	0.169	18.610		4.308	0.741	25.031	
**Sleeping time**					0.1125				0.6625				0.1707				0.8935				0.7539
NA = 61	< 20:00	reference				reference				reference				reference				reference			
	≥ 20:00	0.821	0.644	1.048		0.932	0.674	1.288		0.807	0.592	1.101		0.938	0.309	2.840		1.206	0.335	4.337	
**Waking up**					0.6931				0.8017				0.6903				0.9341				0.8096
NA = 64	≤ 05:00	reference				reference				reference				reference				reference			
	> 05:00	0.951	0.739	1.225		1.042	0.751	1.446		1.067	0.772	1.475		1.040	0.354	3.052		0.867	0.240	3.135	
**Overnight plothut**					**0.0014**				< **0.001**				0.0798				< **0.001**				0.4792
NA = 55	NO	reference				reference				reference				reference				reference			
	YES	1.499	1.165	1.930		1.968	1.390	2.786		1.322	0.958	1.824		7.539	1.357	41.895		1.592	0.354	7.162	
**Overnight forest**					0.3778				0.2161				0.8208				0.8327				0.8543
NA = 62	NO	reference				reference				reference				reference				reference			
	YES	1.150	0.852	1.551		1.272	0.870	1.865		0.955	0.636	1.435		0.877	0.208	3.687		0.865	0.155	4.823	
**Use of bednet**					0.2748				0.1393				0.4405				0.2752				0.0601
NA = 97	NO	reference				reference				reference				reference				reference			
	YES	0.774	0.492	1.218		0.649	0.370	1.138		0.794	0.443	1.423		0.362	0.106	1.242		0.186	0.028	1.215	

OR represents Odds Ratio statistics with respect to the reference category and LCL and UCL represent lower and upper 95% confidence limits based on a total sample size of N = 5793 individuals from 117 villages.

*No *P. malariae* cases were observed in the Khmer Ethnic population.

NA = number of individuals for which the variable was lacking.

Inf = infinity.

The multivariate selection of risk factors confirms the results of the univariate analysis (Table 3). No variables were omitted due to collinearity. The result table of the multivariate analysis highlights the differences in the importance of the age and ethnicity variables between *P. falciparum* and *P. vivax*, where age significantly contributes to the final model of *P. vivax* only and ethnicity to *P. falciparum* only. In addition, the variable overnight stay in the plot hut was significant for all malaria parasites, except for *P. ovale* where the variable use of bed net contributed to the most parsimonious model.

**Table 3:** **Multivariate representation of the selected risk factor associated with malaria prevalence after the AIC model selection procedure**

Malariometric variable		*Plasmodium*( *all*)			*P. falciparum*			*P. vivax*			*P. malariae*			*P. ovale*		
Variable	Level	OR	LCL	UCL	OR	LCL	UCL	OR	LCL	UCL	OR	LCL	UCL	OR	LCL	UCL
**Age (years)**																
	2-5	reference						reference						reference		
	5-14	1.428	0.952	2.143				1.910	1.114	3.277				2.575	0.141	46.889
	15-39	0.966	0.641	1.455				0.978	0.559	1.712				0.838	0.039	17.896
	≥40	0.895	0.571	1.402				0.755	0.401	1.420				0.000	0.000	Inf
**Ethnicity**																
	Khmer	reference			reference											
	Ethnic Minority	2.316	0.797	6.728	2.897	0.642	13.082									
**Overnight plothut**																
	NO	reference			reference			reference			reference					
	YES	1.525	1.173	1.982	1.916	1.340	2.742	1.439	1.028	2.015	6.872	1.527	30.932			
**Use of bednet**																
	NO													reference		
	YES													0.231	0.036	1.471

OR represents Odds Ratio statistics with respect to the reference category and LCL and UCL represent lower and upper 95% confidence limits based on a total sample size of N = 5583 individuals from 117 villages.

### Spatial statistics

**Figure 2:**
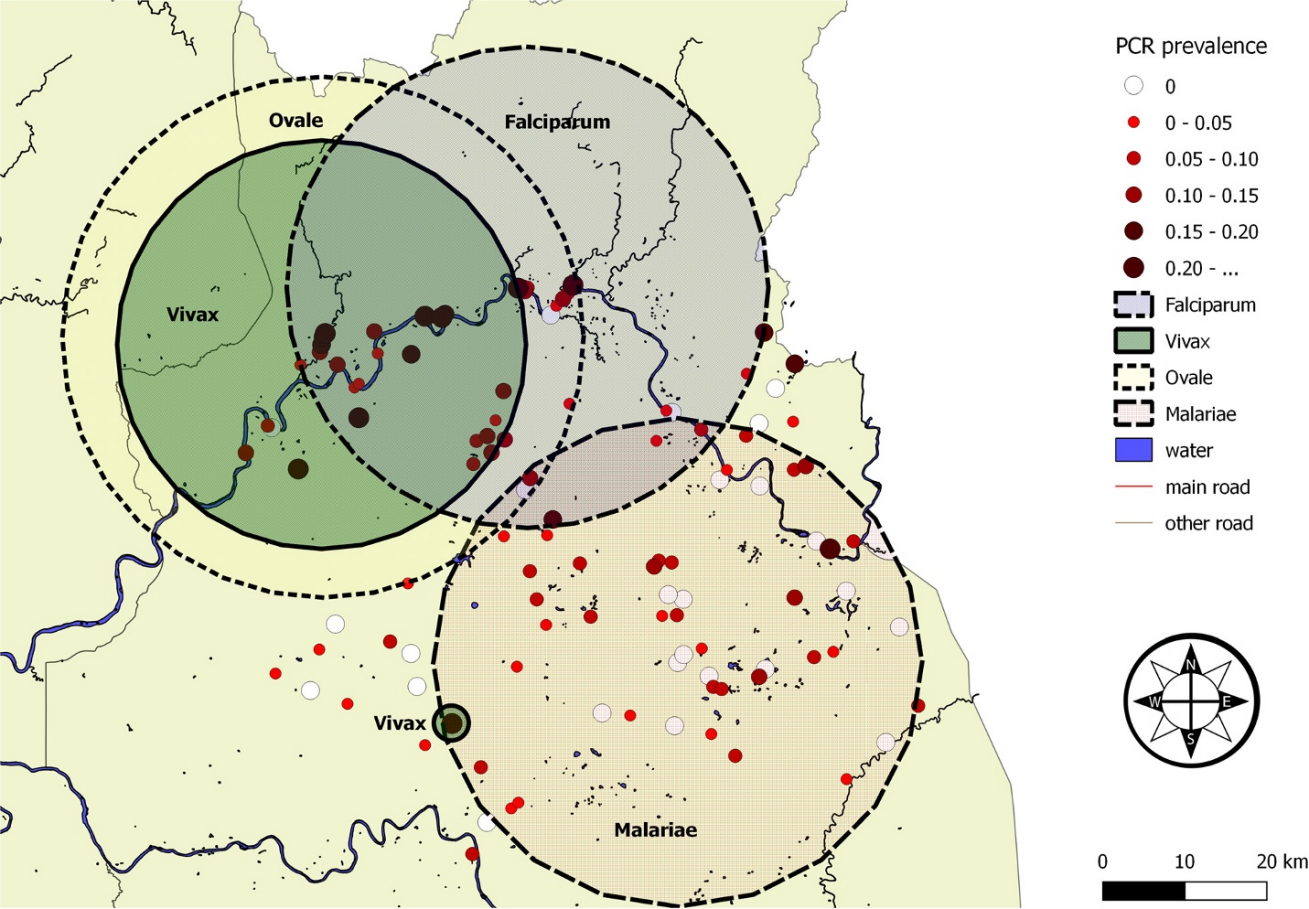
**Map of** ***Plasmodium*** **prevalence in each of the 117 study villages from Ratanakiri province, Cambodia.** Black circles indicate areas of increased risk of infection for the different *Plasmodium* species.

The plot of PCR prevalence of *Plasmodium* spp. per village indicated malaria to be widely distributed in Ratanakiri province (Figure 2). In general our study showed an elevated risk of malaria infection for individuals inhabiting the area along the Sesan river in the north of the province. The SaTScan analysis revealed different areas with a significantly elevated risk for malaria infection (Table 4). For all *Plasmodium* species combined a single cluster was detected along the Sesan river in the north of the province (Observed: 180 cases; Expected: 110, p < 0.0001, cluster not shown on Figure 2). The spatial analysis stratified by *Plasmodium* species indicates areas with an elevated risk of infection to occur at different geographical locations for the different *Plasmodium* species (Figure 2, Table 4). A single cluster along the river in the north was detected for *P. ovale* (Observed cases: 13, Expected cases: 5, p < 0.001) and *P. falciparum* (Observed cases: 124, Expected cases: 85, p < 0.0001). For *P. vivax* an elevated risk was found in two locations, one along the river in the north (Observed cases: 101, Expected cases: 56, p < 0.0001) and one in a single village (Observed cases: 9, Expected cases: 2, p < 0.044). In comparison to the elevated risk areas for *P. falciparum*, *P. vivax* and *P. ovale*, a cluster was detected for *P. malariae* in the south-east of the province (Observed cases: 18, Expected cases: 10, p < 0.039).

**Table 4:** **Spatial clusters of infection with all** ***Plasmodium*** **infection found** (**all**), ***P. falciparum*** (**Pf**), ***P. vivax*** (**Pv**), ***P. malariae*** (**Pm**) **and** ***P. ovale*** (**Po**)

Plasmodium	Clusters	X	Y	Radius in km	Population	Observed	Expected	Relative risk	p-value
all	1	703614	1545020	24,11	1729	180	109,83	2,25	<0,0001
Pf	1	724732	1557240	29,37	2370	124	84,69	2,16	<0,0001
Pv	2	699609	1550260	24,94	1502	101	55,74	2,53	<0,0001
		715521	1504000	0,00*	53	9	1,97	4,73	0,044
Pm	1	743049	1511460	29,81	2711	18	9,83	6,82	0,039
Po	1	699783	1551160	31,79	2306	13	5,17	2,51	0,00061

*A single village was selected as an area with an elevated risk to infection, therefor the radius is 0 km.

### *Plasmodium falciparum*genotyping

**Figure 3:**
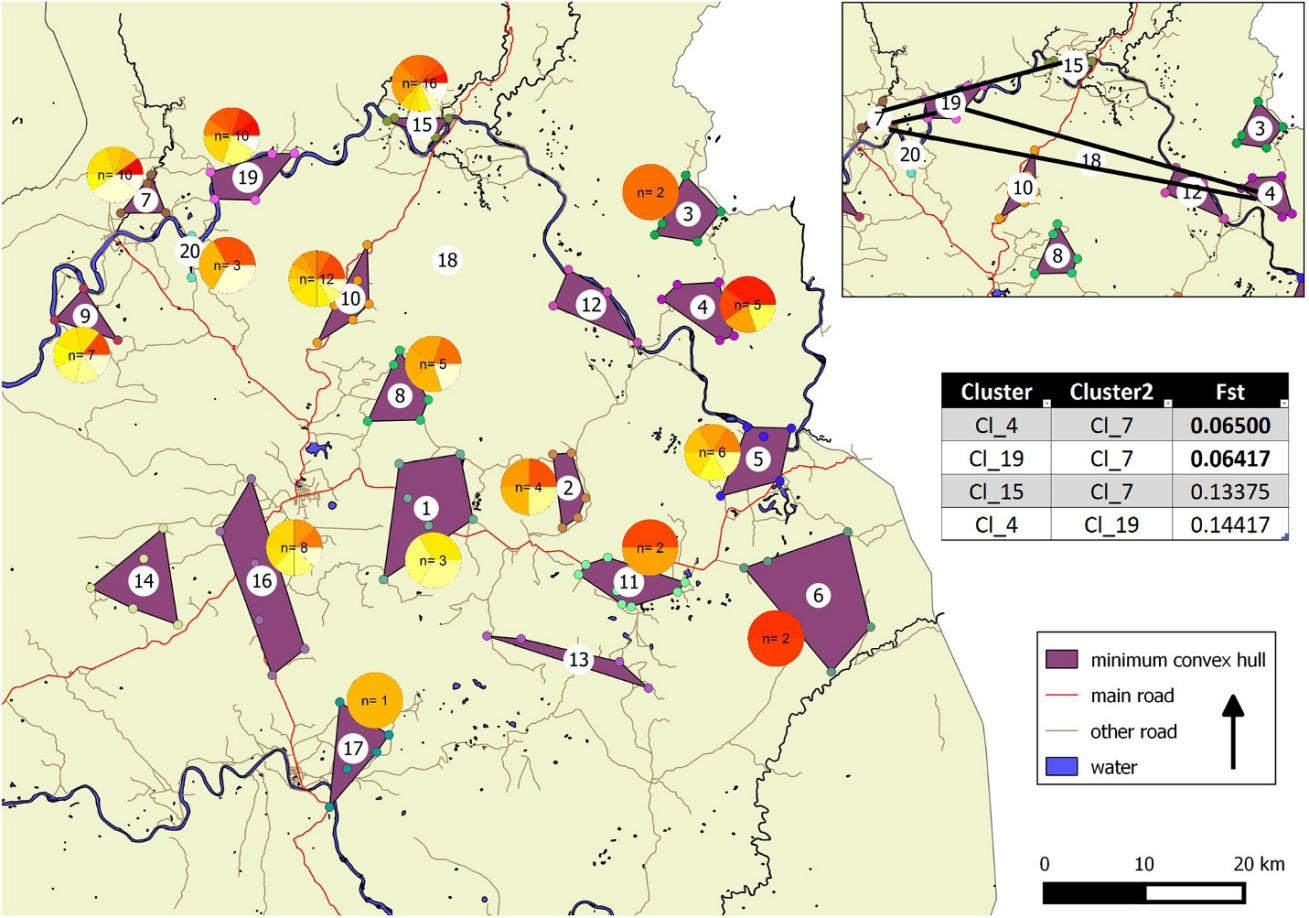
**Overview of the selected clusters for genotyping with indication of the minimum convex hull area and cluster numbers.** Inset figure shows the area along the Sesan river with dark lines connecting clusters with Fst < 0.15. Inset table indicates the Fst values and the respective clusters associated with it. Pie charts, have one color per haplotype and indicate sample size in the center.

Out of the 207 *P. falciparum* infected individuals, 115 (55.6%) had an available barcoding genotype based on 7 SNP’s. For the remaining 92 individuals the parasite density was too low to proceed with the genotyping analysis. Among the 115 genotyped individuals, 19 (16.5%) had polyclonal infections, ten of which belonged to the *Plasmodium falciparum* cluster shown in Figure 2. A total of 43 different haplotypes (34 excluding polyclonal infections) were identified of which 34 (79%) were found in the area with an increased risk for a *P. falciparum* infection and 26 (61%) in the remaining area. Pairwise between cluster Fst values below 0.15, which is an indication of low to moderate genetic differentiation [26], all occurred along the Sesan river (Figure 3, Additional file 3). Pie charts having one colour per haplotype did not indicate geographical pattern of haplotypes grouping together.

## Discussion

Malaria endemic countries that are in the transition progress from control to pre-elimination will benefit from supplementary interventions, targeted towards areas with an increased risk of infection. Cambodia aims to reach the pre-elimination phase in 2015 – 2020 and eliminate malaria by 2025. In 2012, already 13 out of the 24 provinces recorded less than five cases per 1,000 at risk per year, the threshold set by the WHO for entering the pre-elimination phase [3, 7]. However, the current Malaria Information System is based on passive case detection relying on symptomatic cases. Consequently, information regarding the parasite reservoir in asymptomatic populations, which is key knowledge for targeting foci of transmission [27], is presently lacking. The current study provides the first published insights in the spatial occurrence of species-specific clusters of infection, based largely on asymptomatic cases, in the province of Ratanakiri, Cambodia. In combination with the presented risk factor analysis, this information can provide a roadmap for targeting the next wave of interventions in the provinces aiming for pre-elimination.

The spatial aggregation of the *Plasmodium* species resulted in partly overlapping malaria clusters. In the north of the province, along the Sesan river, areas with elevated risks of infection for *P. falciparum*, *P. vivax* and *P. ovale* were detected, with *P. ovale* and *P. vivax* having almost identical cluster centers. An individual living inside the cluster is 2 times more likely to acquire a species-specific *Plasmodium* infection. At a completely different location, in the south-east of the province, an area with an elevated risk for a *P. malariae* infection was identified. These geographical variations could indicate the existence of various species- specific geographical niches, either due to different vector-human interactions or to environmental factors. It has for example been demonstrated that the spatial distribution of G6PD deficiency, widely accepted to be protective against malaria [28], is highly variable in Cambodia [13] or that *Anopheles barbirostris*, recently identified as a secondary vector of *P. malariae* parasites [12], tends to have a different ecological niche as compared to the more sylvatic primary vectors *Anopheles dirus* [29] and *Anopheles miminus* [30].

Both univariate and multivariate risk factor analyses identified age, ethnicity and overnight stay at the plot hut to be significantly associated with the overall malaria prevalence. These variables however were differentially selected for depending on *Plasmodium* species. Age, for example, contributed to the final model predicting *P. vivax* and *P. ovale* infections, but not in the cases of *P. falciparum* and *P. malariae*. As samples were collected during the dry season, a plausible hypothesis is that the relapsing liver stages in both *P. vivax* and *P. ovale* were responsible for this. Indeed, a recent study conducted in the Peruvian Amazon indicated the odds for relapsing liver stages to differ between age groups, but not between sexes [31]. In addition to self-reported overnight stay in the plot hut, ethnicity was identified as a risk factor for *P. falciparum* infections, while the use of a bed net contributed to the *P. ovale* model. The risk factor overnight stay in the plot hut seemed to be the single most important risk factor; reaching statistical significance on a 0.1 level in the univariate analysis in all but one *Plasmodium* species. The multivariate model selection procedure strengthened the importance of this variable as it was again selected for in 3 out of 4 *Plasmodium* species. This result confirmed an additional characteristic to the widely recognized risk groups in the greater Mekong subregion (ethnic minority groups, forest workers and migrants) [15], namely indigenous farming with overnight stays at homes on the field located outside the village. This was also shown among ethnic minority slash-and-burn farmers in Vietnam [32] and in Cambodia in Ratanakiri along the Cambodian-Vietnamese border [33]. In both settings, farmers were more at risk at their fields where they spend a considerable amount of time, including nights, with their families to meet work requirements during the rainy season [32, 34, 35]. In addition, entomological evidence at both localities points towards a higher risk of obtaining infectious bites in the forest as compared to the village [36, 37]. In their conclusion, the authors stated that specific control measures are required targeting families at forest fields in order to protect the most vulnerable populations in economically expanding Southeast Asian societies [32, 34, 35, 37]. Moreover it was observed that farming plots from different villages can share the same location. This would offer a plausible pathway for the parasite to spread from one village to another. Consequently, spatially explicit interventions operated at the village level should take into account this non-migrant but nonetheless mobile behaviour of the resident population, especially in areas where malaria multi-drug resistance occurs.

In contrast to previous studies, the risk factors use of bed net, gender, and overnight stays in the forest were not retained for the final multivariate model. This could be due to the specific context in which the current study was performed. The blood collection took place outside the transmission season, and a widespread bed net distribution campaign ensured the majority of individuals (94%) report to sleep under a bed net. This, in combination with the fact that this study did not differentiate between net use in the village and at the plot huts, could have led to the counterintuitive result of failure to associate the risk factor bed net with malaria in 3 out of 4 species, although the risk of an overall malaria infection was a mere 3% higher for people that indicated not to sleep under a bed net (Additional file 2). Previous studies in Cambodia, conducted during the malaria transmission season, have found males to be associated with an increased risk for *P. falciparum* infection but not for a *P. vivax* infection [38, 39]. The current study showed no association between gender and any of the *Plasmodium* species, and this result seem to confirm that entire families, including both males and females, spend time overnight at their plot huts where they have an increased risk obtaining a malaria infection. Forest activities such as hunting and logging, mainly involving adult males only, are frequently identified as one of the main malaria risks in Cambodia [11, 39]. In the current study the age and gender distribution of individuals which reported to stay overnight in the forest confirms it were mostly adult males. Nevertheless, no association was found between these forest activities and risk of malaria infection. Possibly the fact that this study was conducted in the dry season implies different human and/or mosquito behaviour related to these forest activities. This will be further investigated once data collected in the transmission season within the same project become available.

A high rate of mixed blood stage *Plasmodium* infections (22%) was observed, which in Asia, seemed frequently underreported [40]. This observation is in line with other studies using more sensitive detection methods as compared to standard microscopy [38, 41]. In the present study it was observed that all double co-infections occurred more frequently than expected. This result deviates from a review including 127,000 individuals, where two-way associations were generally found positive on the African continent and negative in Asian countries [17]. In particular the current study provides strong support for a positive association between *P. falciparum* and *P. vivax* in the study area. These contrasting results could partly be explained by the differential detection methods, whereby the studies presented in the review generally used common microscopy to identify different *Plasmodium* species as compared to a more sensitive molecular approach used here [14, 40]. Additionally, infections having low parasite densities, commonly observed for *P. ovale* and *P. malariae* are often missed by classic diagnostic methods such as microscopy [42]. In eastern Cambodia, where severe G6PD deficiency is present in about 5% of the population [13] and where DHA-PIP is the first line of treatment, a species-specific diagnosis is crucial to identify *Plasmodium* species having dormant livers stages. The relapses that can occur when no additional treatment is given for the radical cure of these malaria forms (i.e. primaquine in mild-to-moderate G6PD deficiency) can jeopardize further elimination efforts. In Cambodia, a national guideline for the use of primaquine does exist, but is not yet implemented.

The genotyping of *P. falciparum* did not indicate population structure associated with the area of increased risk to infection. The diversity of the haplotypes in the province seemed to indicate random mixture over all clusters. Only Fst values below 0.15 were observed along the river, which could indicate a moderate tendency for more parasite population flow along the Sesan river. This could potentially be associated with increased movement patterns of infected individuals and/or mosquito populations along this river. However, given that these Fst values are sensitive to sample size these findings need to be confirmed.

## Conclusions

Spatial aggregation of malaria species based largely on asymptomatic infections and the identification of malaria species-specific risk factors provide key insights in malaria epidemiology in low transmission settings. The results of this study will allow targeting future research as well as future interventions aiming at elimination of malaria. The main risk factor associated with infection of three out of four human *Plasmodium* species outside the transmission season in Ratanakiri was overnight stays in plot huts at the farms located outside the village. Consequently, future malaria programmes in the province should implement supplementary strategies, aimed to target households staying overnight at their farms outside the village, in addition to migrants and forest workers.

## Electronic supplementary material

Additional file 1: **Standardized questionnaire.** (DOCX 37 KB)

Additional file 2: **Malariometric data and prevalence per species.** (DOCX 53 KB)

Additional file 3: **Pairwise Fst values between all clusters.** (DOCX 40 KB)
