# Supplementary material for: Spatial clustering and risk factors of malaria infections in Ratanakiri Province, Cambodia
Source: Malar J. 2014 Sep 30;13:387. doi: 10.1186/1475-2875-13-387 (PMC4190307; doi:10.1186/1475-2875-13-387)
Supplement: Supplementary file 1 — Additional file 1: Standardized questionnaire. (DOCX 37 KB) [file 12936_2014_3547_MOESM1_ESM.docx]

**Additional files:**

**Additional file 2:** Malariometric data were gathered on 2 forms for each study participant. On the first form standard malariometric data such as Name, Gender, Age, Fever over the past 48 hours and Axillary Temperature were noted. In the second form following standardized questions were asked.

**To what ethnic group do you belong?**1.Khmer 2.Kreung 3.Tumpourn 4.Charay 5.Prov 6.Cham 7.Kachak 8.Lun 9.Lao 10.Kavet 11.other

**Did you stay overnight in your plot hut at the forest field in the last month?**1:Yes
2:No
3:Don't have plothut
4:Don't remember

**Did you stay overnight in the deep forest in the last month? (ex. Hunting, fishing, collecting wood, etc)**1:Yes
2: No
3: Don't remember

**What time did you usually go to sleep in the evening in the last month?**

**What time did you usually get up in the morning in the last month?**

**What kind of bed net did you sleep in the last month?**1:Bed net given by malaria control program
2:Bed net bought
3:Have not slept under a bed net last month
4:other (specify)_________________
