# Supplementary material for: Spatial clustering and risk factors of malaria infections in Ratanakiri Province, Cambodia
Source: Malar J. 2014 Sep 30;13:387. doi: 10.1186/1475-2875-13-387 (PMC4190307; doi:10.1186/1475-2875-13-387)
Supplement: Supplementary file 2 — Additional file 2: Malariometric data and prevalence per species. (DOCX 53 KB) [file 12936_2014_3547_MOESM2_ESM.docx]

**Additional files:**

**Additional file 1**: Malariometric data and prevalence per species. Single indicate a unique infection (Pf, Pv, Pm or Po) while mixed stands for a combination of two or more malaria species. Note that the % positive per *Plasmodium* species are subtotals calculated as (single+mixed)/Total Sample of each row. NA indicates the amount of missing answers for each specific survey questions. i: outliers were removed.

| **Malariometric Variable** | | ***P. falciparum*** | | | ***P. vivax*** | | | ***P. malariae*** | | | ***P. ovale*** | | | **Total Positive** | **Total Sample** | **Proportion** |
| --- | --- | --- | --- | --- | --- | --- | --- | --- | --- | --- | --- | --- | --- | --- | --- | --- |
|  |  | single | mix | subtotal | single | mix | subtotal | single | mix | subtotal | single | mix | subtotal |  |  |  |
| **Age** | 2-4 | 15 | 5 | 0.032 | 13 | 6 | 0.030 | 1 | 1 | 0.003 | 0 | 1 | 0.002 | 35 | 626 | 0.056 |
| NA = 167 | 5-9 | 22 | 16 | 0.039 | 33 | 17 | 0.051 | 2 | 1 | 0.003 | 0 | 1 | 0.001 | 74 | 980 | 0.076 |
|  | 10-19 | 30 | 26 | 0.041 | 44 | 30 | 0.054 | 2 | 4 | 0.004 | 0 | 9 | 0.007 | 109 | 1376 | 0.079 |
|  | 20-29 | 30 | 5 | 0.036 | 22 | 8 | 0.031 | 1 | 3 | 0.004 | 0 | 2 | 0.002 | 62 | 972 | 0.064 |
|  | 30-39 | 9 | 2 | 0.017 | 9 | 3 | 0.019 | 0 | 0 | 0.000 | 0 | 1 | 0.002 | 21 | 630 | 0.033 |
|  | > 40 | 32 | 11 | 0.037 | 18 | 10 | 0.024 | 2 | 4 | 0.005 | 0 | 0 | 0.000 | 64 | 1174 | 0.055 |
|  |  |  |  |  |  |  |  |  |  |  |  |  |  |  |  |  |
| **Gender** | Males | 68 | 35 | 0.038 | 63 | 38 | 0.037 | 7 | 6 | 0.005 | 0 | 7 | 0.003 | 180 | 2722 | 0.066 |
| NA = 34 | Females | 70 | 30 | 0.033 | 76 | 35 | 0.037 | 1 | 7 | 0.003 | 0 | 7 | 0.002 | 184 | 3037 | 0.061 |
|  |  |  |  |  |  |  |  |  |  |  |  |  |  |  |  |  |
| **Ethnic Minority** | Khmer | 1 | 1 | 0.009 | 1 | 2 | 0.013 | 0 | 0 | 0.000 | 0 | 1 | 0.004 | 4 | 226 | 0.018 |
| NA = 49 | Kroeng | 32 | 17 | 0.045 | 28 | 17 | 0.041 | 3 | 1 | 0.004 | 0 | 2 | 0.002 | 81 | 1091 | 0.074 |
|  | Tumpoun | 23 | 14 | 0.027 | 22 | 13 | 0.026 | 3 | 4 | 0.005 | 0 | 1 | 0.001 | 62 | 1347 | 0.046 |
|  | Charay | 26 | 15 | 0.031 | 24 | 16 | 0.030 | 2 | 6 | 0.006 | 0 | 0 | 0.000 | 70 | 1328 | 0.053 |
|  | Prouv | 32 | 10 | 0.046 | 36 | 14 | 0.054 | 0 | 2 | 0.002 | 0 | 7 | 0.008 | 84 | 923 | 0.091 |
|  | Cham | 1 | 0 | 0.100 | 0 | 0 | 0.000 | 0 | 0 | 0.000 | 0 | 0 | 0.000 | 1 | 10 | 0.100 |
|  | Kachork | 8 | 3 | 0.030 | 7 | 3 | 0.028 | 0 | 0 | 0.000 | 0 | 0 | 0.000 | 18 | 362 | 0.050 |
|  | Lun | 1 | 0 | 0.018 | 4 | 0 | 0.071 | 0 | 0 | 0.000 | 0 | 0 | 0.000 | 5 | 56 | 0.089 |
|  | Lao | 2 | 0 | 0.015 | 3 | 1 | 0.030 | 0 | 0 | 0.000 | 0 | 1 | 0.008 | 6 | 133 | 0.045 |
|  | Kavet | 11 | 7 | 0.067 | 14 | 9 | 0.086 | 0 | 0 | 0.000 | 0 | 2 | 0.007 | 34 | 268 | 0.127 |
|  |  |  |  |  |  |  |  |  |  |  |  |  |  |  |  |  |
| **Fever_48h** | NO | 43 | 21 | 0.032 | 59 | 23 | 0.040 | 3 | 2 | 0.002 | 0 | 4 | 0.002 | 130 | 2027 | 0.064 |
| NA = 95 | YES | 94 | 44 | 0.038 | 80 | 50 | 0.035 | 5 | 11 | 0.004 | 0 | 10 | 0.003 | 233 | 3671 | 0.063 |
|  |  |  |  |  |  |  |  |  |  |  |  |  |  |  |  |  |
| **Temperature** | ≤ 37.5 | 133 | 63 | 0.035 | 136 | 70 | 0.036 | 8 | 13 | 0.004 | 0 | 13 | 0.002 | 353 | 5663 | 0.062 |
| NA = 74 | > 37.5 | 4 | 2 | 0.107 | 3 | 3 | 0.107 | 0 | 0 | 0.000 | 0 | 1 | 0.018 | 10 | 56 | 0.179 |
|  |  |  |  |  |  |  |  |  |  |  |  |  |  |  |  |  |
| **Sleeping timeⁱ** | < 20:00 | 54 | 22 | 0.040 | 62 | 24 | 0.045 | 2 | 5 | 0.004 | 0 | 5 | 0.003 | 144 | 1913 | 0.075 |
| NA = 61 | ≥ 20:00 | 81 | 44 | 0.033 | 76 | 50 | 0.034 | 5 | 8 | 0.003 | 0 | 9 | 0.002 | 216 | 3761 | 0.057 |
|  |  |  |  |  |  |  |  |  |  |  |  |  |  |  |  |  |
| **Waking upⁱ** | ≤ 05:00 | 85 | 29 | 0.036 | 85 | 34 | 0.037 | 4 | 7 | 0.003 | 0 | 8 | 0.002 | 212 | 3204 | 0.066 |
| NA = 64 | > 05:00 | 52 | 37 | 0.035 | 52 | 40 | 0.037 | 3 | 6 | 0.004 | 0 | 6 | 0.002 | 149 | 2516 | 0.059 |
|  |  |  |  |  |  |  |  |  |  |  |  |  |  |  |  |  |
| **Overnight plot** | NO | 42 | 13 | 0.022 | 60 | 15 | 0.030 | 1 | 1 | 0.001 | 0 | 4 | 0.002 | 119 | 2485 | 0.048 |
| NA = 55 | YES | 94 | 54 | 0.045 | 78 | 60 | 0.042 | 7 | 12 | 0.006 | 0 | 10 | 0.003 | 244 | 3253 | 0.075 |
|  |  |  |  |  |  |  |  |  |  |  |  |  |  |  |  |  |
| **Overnight forest** | NO | 109 | 55 | 0.034 | 118 | 61 | 0.037 | 5 | 13 | 0.004 | 0 | 12 | 0.003 | 299 | 4798 | 0.062 |
| NA = 62 | YES | 28 | 12 | 0.043 | 20 | 14 | 0.036 | 3 | 0 | 0.003 | 0 | 2 | 0.002 | 65 | 933 | 0.070 |
|  |  |  |  |  |  |  |  |  |  |  |  |  |  |  |  |  |
| **Used bednet** | NO | 11 | 9 | 0.058 | 8 | 11 | 0.055 | 1 | 2 | 0.009 | 0 | 3 | 0.009 | 31 | 344 | 0.090 |
| NA = 97 | YES | 126 | 57 | 0.034 | 130 | 63 | 0.036 | 6 | 11 | 0.003 | 0 | 11 | 0.002 | 331 | 5352 | 0.062 |
