# Supplementary material for: Spatial clustering and risk factors of malaria infections in Ratanakiri Province, Cambodia
Source: Malar J. 2014 Sep 30;13:387. doi: 10.1186/1475-2875-13-387 (PMC4190307; doi:10.1186/1475-2875-13-387)
Supplement: Supplementary file 3 — Additional file 3: Pairwise Fst values between all clusters. (DOCX 40 KB) [file 12936_2014_3547_MOESM3_ESM.docx]

**Additional files:**

**Additional file 3:** Pairwise standardized Fst values between clusters.

|  | **Cl_2** | **Cl_3** | **Cl_4** | **Cl_5** | **Cl_6** | **Cl_7** | **Cl_8** | **Cl_9** | **Cl_10** | **Cl_11** | **Cl_15** | **Cl_16** | **Cl_17** | **Cl_19** | **Cl_20** |
| --- | --- | --- | --- | --- | --- | --- | --- | --- | --- | --- | --- | --- | --- | --- | --- |
| **Cl_1** | 1.00000 | 0.39292 | 0.48000 | 1.00000 | 1.00000 | 0.23583 | 1.00000 | 1.00000 | 0.97417 | 1.00000 | 0.21958 | 0.82458 | NA | 0.80083 | 1.00000 |
| **Cl_2** |  | 0.46958 | 0.45042 | 1.00000 | 1.00000 | 0.26500 | 1.00000 | 1.00000 | 0.32625 | 1.00000 | 0.16208 | 0.76833 | NA | 0.58917 | 1.00000 |
| **Cl_3** |  |  | 0.24333 | 0.59417 | 0.32958 | 0.17917 | 1.00000 | 0.61375 | 0.82833 | 0.32542 | 0.84417 | 0.35792 | NA | 0.26500 | 0.37958 |
| **Cl_4** |  |  |  | 0.71583 | 0.51792 | 0.06500 | 0.68625 | 0.71375 | 0.73042 | 1.00000 | 0.50750 | 0.48792 | NA | 0.14417 | 0.45292 |
| **Cl_5** |  |  |  |  | 1.00000 | 0.42917 | 1.00000 | 1.00000 | 0.93125 | 1.00000 | 0.85917 | 0.72083 | NA | 0.47333 | 1.00000 |
| **Cl_6** |  |  |  |  |  | 0.33667 | 1.00000 | 1.00000 | 0.50292 | 1.00000 | 0.30333 | 0.64958 | NA | 0.46042 | 1.00000 |
| **Cl_7** |  |  |  |  |  |  | 0.65667 | 0.69208 | 0.31792 | 0.31042 | 0.13375 | 0.47542 | NA | 0.06417 | 0.86917 |
| **Cl_8** |  |  |  |  |  |  |  | 1.00000 | 0.91000 | 1.00000 | 0.75500 | 0.73167 | NA | 0.25167 | 1.00000 |
| **Cl_9** |  |  |  |  |  |  |  |  | 1.00000 | 1.00000 | 0.41625 | 0.91417 | NA | 0.61417 | 1.00000 |
| **Cl_10** |  |  |  |  |  |  |  |  |  | 1.00000 | 0.87417 | 0.64167 | NA | 0.36875 | 0.74042 |
| **Cl_11** |  |  |  |  |  |  |  |  |  |  | 1.00000 | 0.66042 | NA | 0.46125 | 1.00000 |
| **Cl_15** |  |  |  |  |  |  |  |  |  |  |  | 0.32833 | NA | 0.60417 | 0.24250 |
| **Cl_16** |  |  |  |  |  |  |  |  |  |  |  |  | NA | 1.00000 | 0.82208 |
| **Cl_17** |  |  |  |  |  |  |  |  |  |  |  |  |  | NA | NA |
| **Cl_19** |  |  |  |  |  |  |  |  |  |  |  |  |  |  | 0.68458 |
